# Different associations between amyloid-βeta 42, amyloid-βeta 40, and amyloid-βeta 42/40 with soluble phosphorylated-tau and disease burden in Alzheimer’s disease: a cerebrospinal fluid and fluorodeoxyglucose-positron emission tomography study

**DOI:** 10.1186/s13195-023-01291-w

**Published:** 2023-08-30

**Authors:** Caterina Motta, Martina Gaia Di Donna, Chiara Giuseppina Bonomi, Martina Assogna, Agostino Chiaravalloti, Nicola Biagio Mercuri, Giacomo Koch, Alessandro Martorana

**Affiliations:** 1 UOSD Centro Demenze, University of Rome “Tor Vergata”, Rome, Italy; 2 Experimental Neuropsychophysiology Laboratory, IRCCS Santa Lucia Foundation, Rome, Italy; 3 Department of Biomedicine and Prevention, University of Rome Tor Vergata, Rome, Italy; 4 Istituto Neurologico Mediterraneo, Pozzilli, Italy; 5 Human Physiology Unit, Department of Neuroscience and Rehabilitation, University of Ferrara, Ferrara, Italy

**Keywords:** Alzheimer’s disease, Cerebrospinal fluid biomarkers, Amyloid beta 40, Amyloid beta 42, Amyloid beta 42/40 ratio, Phosphorylated-tau, Fluorodeoxyglucose-positron emission tomography

## Abstract

**Background:**

Despite the high sensitivity of cerebrospinal fluid (CSF) amyloid beta (Aβ)_42_ to detect amyloid pathology, the Aβ_42_/Aβ_40_ ratio (amyR) better estimates amyloid load, with higher specificity for Alzheimer’s disease (AD). However, whether Aβ_42_ and amyR have different meanings and whether Aβ_40_ represents more than an Aβ_42_-corrective factor remain to be clarified. Our study aimed to compare the ability of Aβ_42_ and amyR to detect AD pathology in terms of p-tau/Aβ_42_ ratio and brain glucose metabolic patterns using fluorodeoxyglucose-positron emission tomography (FDG-PET).

**Methods:**

CSF biomarkers were analyzed with EUROIMMUN ELISA. We included 163 patients showing pathological CSF Aβ_42_ and normal p-tau (A + T −  = 98) or pathological p-tau levels (A + T +  = 65) and 36 control subjects (A − T −). A + T − patients were further stratified into those with normal (CSFAβ_42_ + /amyR −  = 46) and pathological amyR (CSFAβ_42_ + /amyR +  = 52). We used two distinct cut-offs to determine pathological values of p-tau/Aβ_42_: (1) ≥ 0.086 and (2) ≥ 0.122. FDG-PET patterns were evaluated in a subsample of patients (*n* = 46) and compared to 24 controls.

**Results:**

CSF Aβ_40_ levels were the lowest in A − T − and in CSFAβ_42_ + /amyR − , higher in CSFAβ_42_ + /amyR + and highest in A + T + (*F* = 50.75; *p* < 0.001), resembling CSF levels of p-tau (*F* = 192; *p* < 0.001). We found a positive association between Aβ_40_ and p-tau in A − T − (*β* = 0.58; *p* < 0.001), CSFAβ_42_ + /amyR − (*β* = 0.47; *p* < 0.001), and CSFAβ_42_ + /amyR + patients (*β* = 0.48; *p* < 0.001) but not in A + T + . Investigating biomarker changes as a function of amyR, we observed a weak variation in CSF p-tau (+ 2 *z*-scores) and Aβ_40_ (+ 0.8 *z*-scores) in the normal amyR range, becoming steeper over the pathological threshold of amyR (p-tau: + 5 *z*-scores, Aβ_40_: + 4.5 *z*-score). CSFAβ_42_ + /amyR + patients showed a significantly higher probability of having pathological p-tau/Aβ_42_ than CSFAβ_42_ + /amyR − (cut-off ≥ 0.086: OR 23.3; cut-off ≥ 0.122: OR 8.8), which however still showed pathological values of p-tau/Aβ_42_ in some cases (cut-off ≥ 0.086: 35.7%; cut-off ≥ 0.122: 17.3%) unlike A − T − . Accordingly, we found reduced FDG metabolism in the temporoparietal regions of CSFAβ_42_ + /amyR − compared to controls, and further reduction in frontal areas in CSFAβ_42_ + /amyR + , like in A + T + .

**Conclusions:**

Pathological p-tau/Aβ_42_ and FDG hypometabolism typical of AD can be found in patients with decreased CSF Aβ_42_ levels alone. AmyR positivity, associated with higher Aβ_40_ levels, is accompanied by higher CSF p-tau and widespread FDG hypometabolism.

**Supplementary Information:**

The online version contains supplementary material available at 10.1186/s13195-023-01291-w.

## Background

According to the amyloid cascade hypothesis, Alzheimer’s disease (AD) develops due to amyloid peptides (Aβ) deposition in senile plaques, followed by the accumulation of hyperphosphorylated tau proteins (p-tau) in tangles, ultimately leading to neuronal degeneration and cognitive decline. In 2018, the National Institute on Aging-Alzheimer’s Association (NIA-AA) reported that the highest probability of identifying AD pathology in vivo is achieved by combining markers of Aβ (A) and p-tau (T) pathology [1]. Indeed, it has been demonstrated that the co-presence of A + and T + status, as well as combining them in the CSF p-tau/Aβ_42_ ratio, provides excellent predictive power for the presence of AD pathology at autopsy [2, 3]. However, when there is no concordance between A and T, and notably when the CSF levels of Aβ_42_ are abnormal (A +) without concomitant abnormal values of p-tau (T −), we encounter an ambiguous biological profile, labeled “AD pathological changes,” which is still considered part of the Alzheimer’s continuum. Given the crucial role of the “A” status in this biological condition, inconsistencies among different amyloid biomarkers, from cerebrospinal fluid (CSF) or imaging, can represent a significant limitation in supporting a possible diagnosis of AD, thus fueling the need to improve their accuracy.

In this regard, it has been demonstrated that the addition of CSF Aβ_40_ to CSF Aβ_42_ levels in the Aβ_42_/Aβ_40_ ratio (amyR) could account for inter-individual variations in Aβ production, thereby enhancing the diagnostic performance of this biomarker [4–8]. Furthermore, amyR seems to better reflect the total amount of amyloid brain deposition [9–12]. Nevertheless, Vromen and colleagues also found that the decrease of CSF Aβ_42_ alone shows excellent accuracy in detecting autopsy-confirmed AD [13]. Together, these data support the idea that amyR and Aβ_42_ may provide different kinds of information on the underlying AD pathophysiology.

In the present study, we aimed to determine whether CSF Aβ_40_ has a specific meaning, which enables amyR to better estimate the AD-related burden, and whether pathological CSF Aβ_42_ in the presence of normal amyR can still identify AD pathology. To address this issue, we focused on the A + T − biological condition, stratifying patients with decreased CSF Aβ_42_ into those with normal (CSFAβ_42_ + /amyR −) or pathological amyR (CSFAβ_42_ + /amyR +). We assessed differences and overlaps with healthy control subjects (A − T −) and full-blown AD patients (A + T +) in terms of disease burden measured as CSF p-tau/Aβ_42_ [2, 14] and cerebral glucose hypometabolism, evaluated with fluorodeoxyglucose-positron emission tomography (FDG-PET), whose specific topographical patterns have achieved an increasingly supportive role in the diagnostic algorithm of AD [15–17].

## Materials and methods

### Subjects’ enrolment

Between 2017 and 2021, we evaluated 250 patients at the Memory Clinic of the “Policlinico Tor Vergata” in Rome. The criteria for retrospective inclusion were as follows: (1) a complete diagnostic workup, including standardized neurological examination, laboratory testing, MRI imaging, FDG-PET scan, neuropsychological assessment, APOE genotyping, and CSF analysis, and (2) fulfillment of the diagnostic criteria for dementia [18] or mild cognitive impairment due to AD [19]. Exclusion criteria were as follows: (1) presence of other neurological or psychiatric diseases or medical conditions potentially associated with cognitive deficits; (2) major comorbidities such as oncological history, systemic inflammatory conditions, and organ failure; (3) prominent cortical or subcortical infarcts; (4) history of drug or alcohol abuse; (5) use of antipsychotics, antidepressants, or serotonergic drugs.

Eventually, we selected 163 patients belonging to the Alzheimer’s continuum (ADc) according to their biomarker profile [1]. Further stratification into AT groups was performed according to the presence of decreased CSF levels of Aβ_42_ (A) and increased CSF p-tau (T) (see Additional file 1).

Furthermore, we identified 36 controls among inpatients from the Neurology Unit of Policlinico Tor Vergata who had undergone a complete neurological evaluation, brain CT, and lumbar puncture for diagnostic purposes and for which the presence of any primary neurological disease had been excluded. All CSF analyses showed normal cell counts and biomarker profiles.

### CSF sampling/analysis and APOE genotyping

All lumbar punctures were performed between 8 and 10 am. An 8 ml CSF sample was collected for each patient in polypropylene tubes, 2 ml were used for routine biochemical analysis, 6 ml were centrifuged at 2000 g at + 4 °C for 10 min, and frozen at – 80 °C. All samples were processed according to the manufacturer’s instructions and to laboratory standard operating procedures.

The levels of CSF Aβ_42_ and Aβ_40_, t-tau, and p-tau phosphorylated at Thr181 (p-tau181) were determined using a sandwich enzyme-linked immunosorbent assay (EUROIMMUN ELISA©). The cut-off values for CSF Aβ_42_, CSF p-tau, and CSF t-tau were determined following EUROIMMUN guidelines: CSF Aβ_42_ > 600 pg/ml, CSF amyR > 0.06, CSF p-tau < 65 pg/ml, CSF t-tau < 400 pg/ml. Given the absence of manufacturer’s guidelines for the p-tau/Aβ_42_ cut-off and of neuropathological/amyloid PET imaging validation in literature, we performed our analyses on AD-related burden (p-tau/Aβ_42_) considering two separate thresholds to determine pathological values: (1) ≥ 0.086, determined with a similar ELISA technique (INNOTEST©) and validated on neuropathological data [2], and (2) ≥ 0.122, determined on EUROIMMUN assays with a Youden Index analysis to discriminate between biologically defined AD and non-AD patients [20].

APOE genotyping was performed using allelic discrimination technology with real-time PCR (TaqMan; Applied Biosystems). Patients were classified as APOE4 when carrying either one (APOE ε3/ε4) or two (APOE ε4/ε4) ε4 alleles. All the remaining patients were identified as APOE3 (ε3/ε3).

### FDG-PET substudy

#### Study population

The study was conducted at the Nuclear Medicine Unit of Policlinico Tor Vergata in Rome (General Electric VCT PET/CT scanner). Among the enrolled patients, 46 had undergone FDG-PET at our site (CSFAβ42 + /amyR − , *n* = 9; CSFAβ42 + /amyR + , *n* = 17; A + T + , *n* = 20).

Moreover, 24 subjects (male, 10; female, 14; mean age, 66.56 ± 10.44 years) undergoing FDG-PET/CT for other reasons were enrolled as part of a control group (CG) upon showing no signs of hypometabolism suggestive of neurodegenerative disorders nor other cerebral abnormalities, as assessed by visual reads (A.C). All subjects had an MRI performed within 14 ± 4 days before PET/CT examination, showing absence of brain alterations. An experienced neurologist (A.M.) evaluated all participants to assess the absence of clinical signs of cognitive decline. Patient and control selection strategies for the sub-study are summarized in Additional file 2.

#### FDG-PET/CT scanning and acquisition

The same scanning and acquisition protocol was used for both patients and CG. All subjects were injected with intravenous FDG (dose range 185–295 megabecquerels) and hydrated with 500 ml of saline (0.9% sodium chloride). PET/CT acquisition started 30 ± 5 min after FDG injection and lasted for 10 min in all subjects. The reconstruction parameters were as follows: ordered subset expectation maximization, four subsets and 12 iterations; matrix 256 × 256; full width at half maximum (FWHM): 5 mm.

### Data management and statistical analysis

#### CSF biomarkers analysis

All continuous variables were expressed as mean ± standard deviation. The Kruskal–Wallis test and Dunn’s post hoc analysis were used for multiple comparisons. Categorical variables were analyzed using Pearson’s chi-square test. We computed a linear regression model with age and sex as covariates to study the association between CSF amyloid biomarkers and CSF p-tau levels. We then performed a robust locally weighted regression analysis [21] and plotted CSF biomarkers levels as a function of amyR, using CSF biomarkers values converted to z-scores by subtracting the mean and dividing by the standard deviation of the control group (A − T −). Eventually, logistic multivariate regressions were performed to evaluate the odds of pathological p-tau/Aβ_42_ values according to amyR status, accounting for clinical and demographic factors (age, sex, APOE, diabetes mellitus, hypertension, dyslipidemia).

All statistical analyses were performed using StataCorp© (Stata Statistical Software: Release 13. College Station, TX: StataCorp) and GraphPad Prism version 9.3.1 (GraphPad Software, San Diego, California, USA). All results were computed with two-tailed significance; *p* < 0.05 were considered significant.

#### FDG-PET analysis

First, all FDG-PET scans were visually evaluated in standardized transaxial, coronal, and sagittal plans by an experienced nuclear medicine physician (A.C) and interpreted according to the latest EANM guidelines for FDG-PET imaging [22]. We used Statistical Parametric Mapping 12 (SPM12) in MATLAB 2018a (https://www.fil.ion.ucl.ac.uk/spm/software/spm12/) to perform statistical analysis. FDG-PET data were converted from DICOM to Nifti format using Mricron software (https://www.nitrc.org/projects/mricron) and then subjected to a normalization process. A bias regularization was applied (0.0001) to limit biases due to smoothness, spatially varying artifacts that modulate the intensity of the image and that can impede automating processing of images. FWHM of Gaussian smoothness of bias (to prevent the algorithm from trying to model out intensity variation due to different tissue types) was set at 60 mm cut-off; tissue probability map implemented in SPM12 was used (TPM.nii). A mutual information affine registration with the tissue probability maps [23] was used to achieve approximate alignment to ICBM space template—European brains [24, 25]. Warping regularization was set with the following 1 by 5 array (0, 0.001, 0.5, 0.05, 0.2); smoothness (to cope with functional anatomical variability that is not compensated by spatial normalization and to improve the signal-to-noise ratio) was set at 5 mm; sampling distance (that encodes the approximate distance between sampled points when estimating the model parameters) was set at 3. We applied an 8-mm isotropic Gaussian filter to blur the individual variations (especially gyral variations) and to increase the signal-to-noise ratio. We used the following parameters and post-processing tools before regression analysis was applied: global normalization (that scales images to a global value) = 50 (using proportional scaling); masking threshold (that helps to identify voxels with an acceptable signal in them) was set to 0.8; transformation tool of statistical parametric maps into normal distribution; correction of SPM coordinates to match the Talairach coordinates, subroutine implemented by Matthew Brett (http://www.mrc-cbu.cam.ac.uk/Imaging). Brodmann areas (BA) were identified at a range from 0 to 3 mm from the corrected Talairach coordinates of the SPM output isocenter, using a Talairach client available at http://www.talairach.org/index.html. As proposed by Bennett et al. [26], SPM t-maps were corrected for multiple comparisons using the false discovery rate (*p* ≤ 0.05) and corrected for multiple comparisons at the cluster level (*p* ≤ 0.001). The level of significance was set at 100 contiguous voxels (5 × 5 × 5 voxels, i.e., 11 × 11 × 11 mm). The following voxel-based comparisons were assessed: CG vs. CSFAβ_42_ + /amyR − , CSFAβ_42_ + /amyR − vs. CSFAβ_42_ + /amyR + , and CSFAβ_42_ + /amyR + vs. A + T + . We used a full factorial design implemented in SPM12 to test the hypothesis that differences among groups exist overall. Comparisons between groups were performed using the two-sample *t*-test model of SPM12. For both analyses, age and sex were used as covariates, and the threshold was set at *p* < 0.001 (*p* < 0.05 FWE corrected at the cluster level).

## Results

### Participants’ selection and characteristics

From 250 subjects with suspected AD-related cognitive impairment, we enrolled 163 patients with decreased CSF levels of Aβ_42_ and normal CSF p-tau (A + T −  = 98) or pathological CSF p-tau (A + T +  = 65), as well as 36 sex-/age-matched healthy controls (A − T −) (see Additional file 1). We further stratified A + T − patients according to the presence of either pathological (CSFAβ_42_ + /amyR + , *n* = 46) or normal amyR (CSFAβ_42_ + /amyR − , *n* = 52). Table 1 shows the clinical and demographic characteristics of the patients according to AT status.

**Table 1:** Demographic and clinical characteristics across AT groups

	**A − T − (*n* = 36)**	**CSFAβ**_**42**_** + /amyR − (*n* = 52)**	**CSFAβ**_**42**_** + /amyR + (*n* = 46)**	**A + T + (*n* = 65)**	***p***
Age (years)	70.72 ± 3.81	68.90 ± 9.55	71.34 ± 8.91	72.31 ± 6.56	0.15
Male (%)	33.3%	55.7%	60.8%	33.8%	** < 0.01**
CSF Aβ_42_ (pg/ml)	1270.91 ± 196.67	394.45 ± 85.80	341.92 ± 81.18	389.17 ± 105.41	** < 0.001**
CSF Aβ_40_ (pg/ml)	5870.36 ± 1283.07	4740.74 ± 1457.22	8110.55 ± 2490.97	10092.15 ± 3352.14	** < 0.001**
CSF p-tau (pg/ml)	25.27 ± 6.65	29.12 ± 13.63	44.84 ± 13.28	101.37 ± 30.89	** < 0.001**
CSF t-tau (pg/ml)	165.82 ± 60.60	149.30 ± 77.60	233.84 ± 72.53	646.54 ± 261.38	** < 0.001**
CSF p-tau/Aβ_42_	0.02 ± 0.01	0.07 ± 0.04	0.14 ± 0.05	0.28 ± 0.11	** < 0.001**
Qalb	5.62 ± 3.40	8.75 ± 6.33	7.46 ± 4.11	7.44 ± 6.58	** < 0.01**
MMSE	29.19 ± 1.97	21.31 ± 4.82	21.50 ± 5.15	20.25 ± 4.95	** < 0.001**
MCI/Dementia (*n*)	0/0	21/31	18/28	15/50	0.08
Diabetes (%)	38.9%	55.8%	56.5%	58.5%	0.26
Hypertension (%)	47.2%	21.2%	19.6%	15.4%	** < 0.01**
Dyslipidemia (%)	27.8%	19.2%	26.1%	35.4%	0.28
APOE4 (%)	n.a	28.8%	32.6%	55.4%	** < 0.01**

Data are presented as mean ± standard deviation or percentages, when applicable. *CSF* cerebrospinal fluid, *Qalb* albumin quotient, *MMSE* Mini-Mental State Examination, *p p*-value. MMSE score of ≥ 24 was used as a cut-off to separate MCI from dementia patients. Percentages indicate subjects showing the presence of each variable within the group. Bold values represent *p*-value < 0.05

### CSF AD biomarkers and disease burden

CSF levels of Aβ_42_ were significantly lower in CSFAβ_42_ + /amyR − , CSFAβ_42_ + /amyR + , and A + T + patients than in A − T − subjects (*F* = 560.9; *p* < 0.001), but no differences were found among the patient groups (*p* > 0.05). Conversely, CSF Aβ_40_ levels were similarly lower in A − T − and CSFAβ_42_ + /amyR − , significantly higher in CSFAβ_42_ + /amyR + , and even higher in A + T + patients (*F* = 50.75; *p* < 0.001), suggesting that pathological amyR in CSFAβ_42_ + /amyR + and A + T + could be sustained by the concomitant presence of increased Aβ_40_ and decreased Aβ_42_ in the CSF. Similarly, the ANOVA showed lower CSF p-tau levels in A − T − and CSFAβ_42_ + /amyR − , higher in CSFAβ_42_ + /amyR + , and highest in A + T + (*F* = 192; *p* < 0.001).

**Fig. 1:**
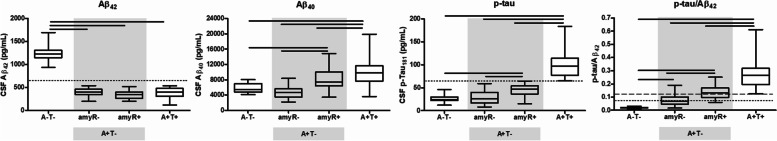
Intergroup differences of cerebrospinal fluid (CSF) biomarkers. The gray zone includes patients classified as A + T − . Dotted lines represent cut-off values used for Aβ_42_, p-tau, and the p-tau/Aβ_42_ cut-off 0.086; the dashed line represents p-tau/Aβ_42_ cut-off 0.122. Bold lines represent comparisons with *p*-values < 0.05 at the Kruskal–Wallis

Finally, p-tau/Aβ_42_ levels were the lowest in A − T − and progressively increased in CSFAβ_42_ + /amyR − , CSFAβ_42_ + /amyR + , and A + T + patients (*F* = 131.9; *p* < 0.001) (Fig. 1).

### Association between amyloid biomarkers and p-tau CSF levels

To explore the association between different CSF amyloid biomarkers (Aβ_42,_ Aβ_40_, and amyR) and CSF p-tau levels, we performed correlation (see Additional file 3) and linear regression analyses adjusting for age and sex (see Table 2). Aβ_42_ was associated with p-tau levels only in A − T − subjects (*β* = 0.48; *p* = 0.003), but not in any other AT subgroup. In contrast, a positive association between Aβ_40_ and CSF p-tau levels was found in A − T − (*β* = 0.58; *p* < 0.001), CSFAβ_42_ + /amyR − (*β* = 0.47; *p* < 0.001), and CSFAβ_42_ + /amyR + patients (*β* = 0.48; *p* < 0.001), but not in A + T + patients. Similarly, amyR was associated with CSF p-tau levels in A − T − (*β* =  − 0.33; *p* = 0.047), CSFAβ_42_ + /amyR − (*β* =  − 0.49; *p* < 0.001), and CSFAβ_42_ + /amyR + patients (*β* =  − 0.45; *p* < 0.001), but not in A + T + patients.

**Table 2:** Linear regression analyses, adjusted for age and sex, assessing the association between CSF amyloid biomarkers and CSF levels of p-tau

	A − T −		CSFAβ_42_ + /amyR −		CSFAβ_42_ + /amyR +		A + T +	
	*β*	*p*	*β*	*p*	*β*	*p*	*β*	*p*
Aβ_42_	**.48**	**.003**	.10	.44	.12	.33	.16	.20
Aβ_40_	**.58**	** < .001**	**.47**	** < .001**	**.48**	** < .001**	− 0.07	.58
amyR	** − .33**	**.047**	** − .49**	** < .001**	** − .45**	** < .001**	.23	.08

Bold values represent *p*-value < 0.05

*amyR* Aβ_42_/Aβ_40_, *β* standardized coefficient, *p p*-value

**Fig. 2:**
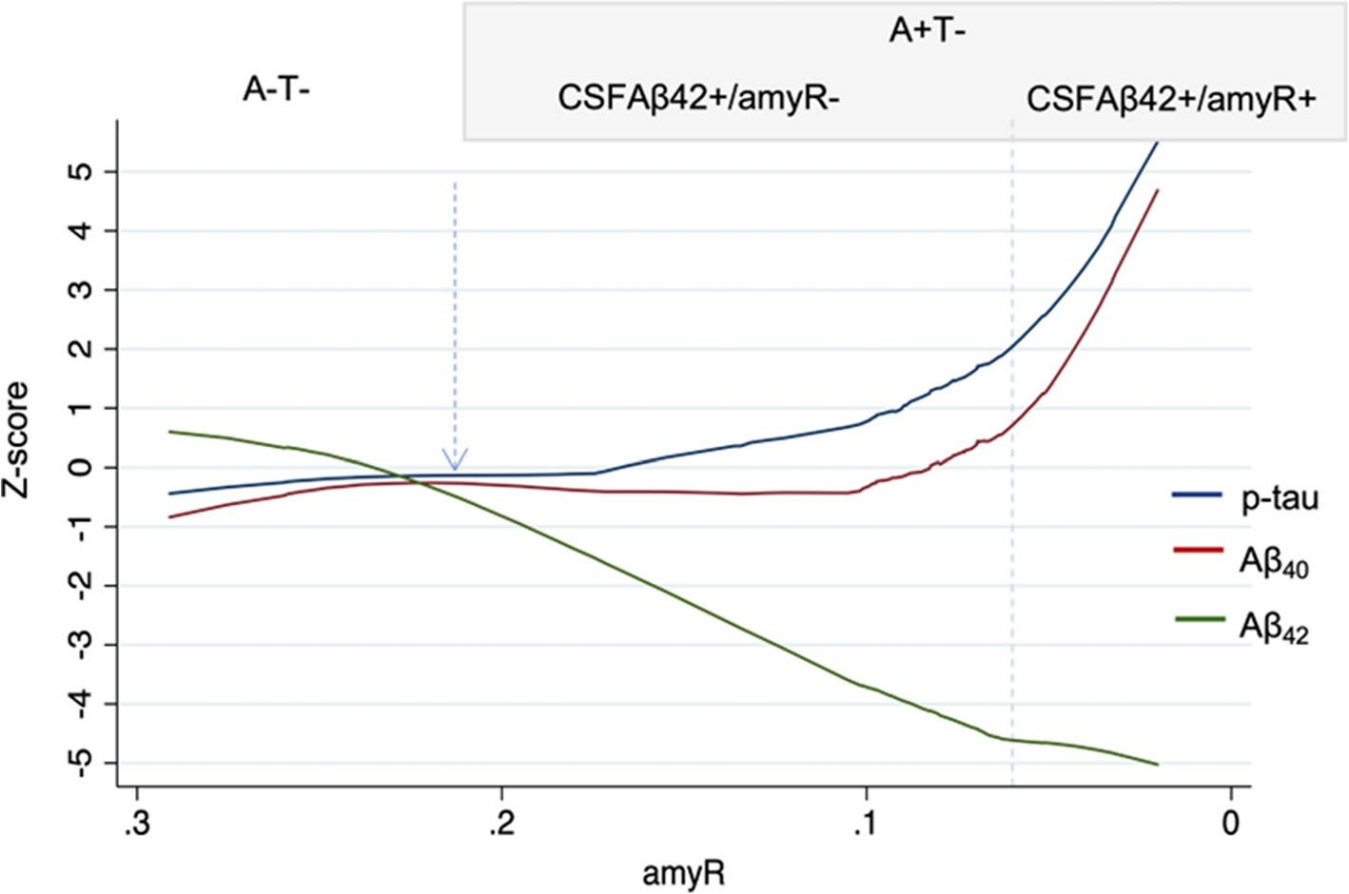
Results of the robust locally weighted regression analysis showing changes of CSF biomarkers as a function of amyR. Values of *p*-tau, Aβ_42_, and Aβ_40_ are expressed as *z*-score standardized on the average and standard deviation of the A − T − group. The dotted line represents threshold value of pathological amyR (0.06), the dotted arrow indicates the point of change of Aβ_42_ from normal to pathological values. CSF, cerebrospinal fluid; amyR, Aβ_42_/Aβ_40_; A, amyloid status; T, p-tau status)

Given the previous association between amyloid and p-tau markers in the A − T − and A + T − subgroups, we applied a robust locally weighted regression model to trace the trajectories of standardized (z-scores) CSF biomarkers as a function of amyR. We observed that CSF Aβ_42_ dramatically declined, reaching − 4.5 *z*-scores, when amyR levels were still normal, and plateaued when amyR became pathological (− 5 *z*-scores). Remarkably, CSF Aβ_40_ and p-tau also started to increase before the amyR cut-off was reached (2 *z*-scores for CSF p-tau and 0.8 *z*-scores for Aβ_40_), but steeply increased above the pathological threshold of amyR, eventually reaching the highest z-scores (5 *z*-scores for CSF p-tau and 4.5 *z*-scores for Aβ_40_) (Fig. 2).

### Pathological amyR increases odds of pathological p-tau/Aβ_42_ in A + T − 

To evaluate the effect of amyR on the AD-related burden, in terms of p-tau/Aβ_42_ ratio, we applied Pearson’s chi-squared test to A + T − patients and found a different distribution of pathological p-tau/Aβ_42_ levels between CSFAβ_42_ + /amyR − and CSFAβ_42_ + /amyR + subgroups [cut-off ≥ 0.086: 35.7% vs 78%, *χ*^2^ (1, *n* = 105) = 27.018, *p* < 0.001; cut-off ≥ 0.122: 17.3% vs 63%, *χ*^2^ (1, *n* = 105) = 21.506, *p* < 0.001].

In the logistic multivariate analyses, considering the variables age, sex, APOE E4 allele, hypertension, diabetes, and dyslipidemia, pathological amyR was significantly associated with a higher likelihood of having pathological p-tau/Aβ_42_ [cut-off ≥ 0.086: OR 23.3 with a 95% CI of 4.0980–28.4627, *p* < 0.001; cut-off ≥ 0.122: OR 8.8 with a 95% CI of 3.28–23.68, *p* < 0.001] (Table 3). Nevertheless, some patients with normal amyR in the CSFAβ_42_ + /amyR − group still showed pathological p-tau/Aβ_42_ levels (cut-off ≥ 0.086: 35.7%; cut-off ≥ 0.122: 17.3%).

**Table 3:** Results of multivariate regression analysis exploring the odds of having pathological values of p-tau/Aβ_42_ in A + T − patients

	**p-tau/Aβ**_**42**_** cut-off ≥ 0.086**		**p-tau/Aβ**_**42**_** cut-off ≥ 0.122**	
	**Odds ratio (CI)**	***p***	**Odds ratio (CI)**	***p***
**amyR**	23.27 (6.47–83.60)	** < 0.001**	8.82 (3.28–23.68)	** < 0.001**
**Diabetes**	0.38 (0.11–1.32)	0.13	0.51 (0.19–1.40)	0.19
**Hypertension**	0.99 (0.26–3.72)	0.99	1.25 (0.39–4.03)	0.71
**Dyslipidemia**	2.79 (0.71–10.95)	0.14	1.09 (0.34–3.47)	0.88
**APOE4**	5.93 (1.61–21.79)	**0.007**	1.27 (0.45–3.58)	0.65
**Age**	1.07 (0.99–1.14)	0.06	1.02 (0.96–1.07)	0.48
**Sex**	1.31 (0.43–4.03)	0.63	1.82 (0.68–4.91)	0.23

Bold values represent *p*-value < 0.05

*amyR* Aβ_42_/Aβ_40_, *CI* 95% confidence interval, *p p*-value

### FDG-PET substudy

Demographics of the participants to the substudy are reported in Additional file 4. Two expert raters (A.M. and A.C.) separately evaluated FDG-PET scans from the 46 patients in the substudy and identified 37 typical AD patterns, four possible AD patterns, and five normal scans, showing good inter-rater agreement (Cohen’s *k* = 0.9). Holding scans with both typical and possible patterns as AD-positive, Cohen’s *k* analysis demonstrated a significant agreement between abnormal FDG-PET and pathological CSF p-tau/Aβ_42_ (Cohen’s *k* = 0.73), with a mismatch of 6% between them.

**Fig. 3:**
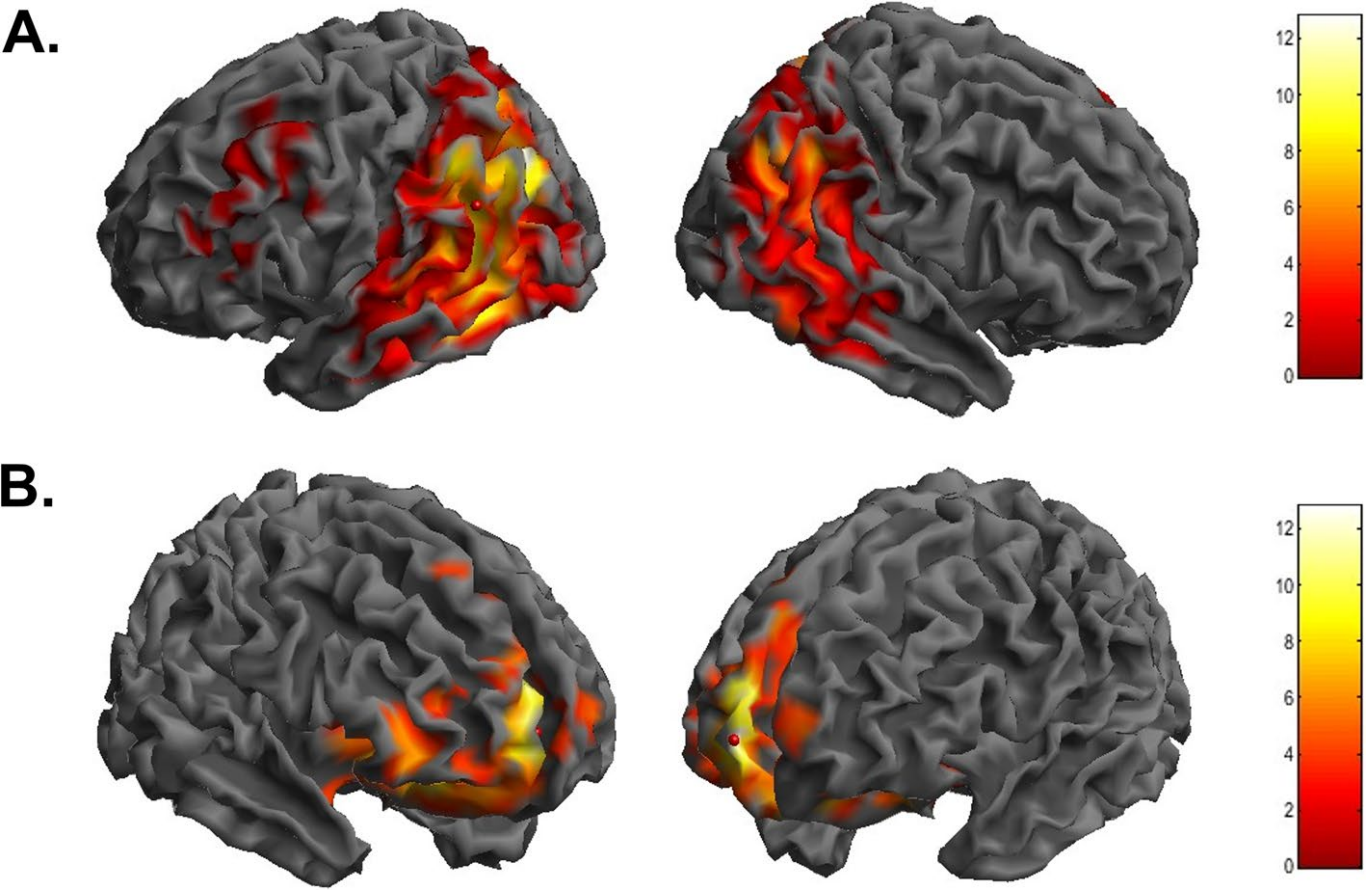
3D brain rendering showing significant clusters obtained in SPM when comparing A − T − vs. CSFAβ_42_ + /amyR − (**A**) and CSFAβ_42_ + /amyR − vs. CSFAβ_42_ + /amyR + (**B**). Color scale represents *t*-statistics values

Comparisons between FDG-uptake patterns of each patient group versus CG showed a significant reduction in the temporo-parietal and frontal regions (see Additional file 5). The full factorial design, accounting for inter-group differences, resulted in a significant main effect of groups, and results from the post hoc analysis are shown in Table 4. Compared to the control group (CG), CSFAβ_42_ + /amyR − patients showed a significant reduction in brain glucose consumption in a wide cluster encompassing the left parietal (BAs 19 and 40), temporal (BA 37), and frontal lobes (BAs 6, 8, and 46) (Fig. 3A). With respect to CSFAβ_42_ + /amyR − , CSFAβ_42_ + /amyR + showed an additional reduction in FDG uptake in the anterior regions, namely the anterior cingulate and frontomedial areas (BAs 32 and 10) (Fig. 3B). No differences were found between the hypometabolic patterns of CSFAβ_42_ + /amyR + and A + T + patients (*p* > 0.05).

**Table 4:** Numerical results of SPM comparisons of FDG uptake in CG vs. CSFAβ_42_ + /amyR − , CSFAβ_42_ + /amyR − vs. CSFAβ_42_ + /amyR + , and CSFAβ_42_ + /amyR + vs. A + T +

Analysis	Cluster level					Voxel level		
	**Cluster *p* (FWE-corr)**	**Cluster *p* (FDR-corr)**	**Cluster extent**	**Cortical region**	***Z* score of maximum**	**Talairach coordinates**	**Cortical region**	**BA**
**CG vs CSFAβ**_**42**_** + /amyR − **	0.000	0.000	31,175	L parietal lobe	Inf	− 36, − 74, 38	Precuneus	19
				L temporal lobe	Inf	− 52, − 56, − 10	Middle temporal gyrus	37
				L parietal lobe	7.63	− 48, − 48, 44	Inferior parietal lobule	40
	0.001	0.000	1795	L frontal lobe	3.81	− 26, 10, 58	Middle frontal gyrus	6
				L frontal lobe	3.61	− 30, 26, 46	Middle frontal gyrus	8
				L frontal Lobe	3.43	− 48, 30, 16	Inferior frontal gyrus	46
**CSFAβ**_**42**_** + /amyR − vs CSFAβ**_**42**_** + /amyR + **	0.000	0.000	6851	R frontal lobe	5.06	14, 62, 0	Medial frontal gyrus	10
				R frontal lobe	4.63	8, 60, 14	Medial frontal gyrus	10
				R limbic lobe	4.58	16, 32, 18	Anterior cingulate	32
**CSFAβ**_**42**_** + /amyR + vs A + T + **	n.s			n.a	n.a	n.a	n.a	n.a

In the “cluster level” section (left), the number of voxels, corrected *p*-value of significance, and cortical region where the voxel is found are all reported for each significant cluster. In the “voxel level” section, all the coordinates of the correlation sites (with the *Z*-score of the maximum correlation point), the corresponding cortical region and BA are reported for each significant cluster (CG, control group; Inf., infinite; L, left; R, right; BA, Brodmann area). When the maximum correlation is achieved outside the gray matter, the nearest gray matter (within a range of 5 mm) is indicated by the corresponding BA

## Discussion

In the complex AD framework, the discordance between biomarkers, especially those accounting for amyloid status, might encourage both clinicians and researchers to prefer biomarkers with higher specificity, despite the risk of losing sensitivity and likely missing the problem of the different meanings associated with those biomarkers.

In the present study, we investigated whether Aβ_42_ and amyR have different associations with AD-related burden, measured by CSF p-tau/Aβ_42_ [2, 14, 20] and FDG-PET brain hypometabolism.

### Main findings in CSF AD biomarkers and disease burden

CSF Aβ_42_ levels did not significantly change across the AD continuum. Conversely, we identified a stepwise increase in CSF Aβ_40_, with lower levels in controls as in CSFAβ_42_ + /amyR − , higher levels in CSFAβ_42_ + /amyR + , and the highest in A + T + , resembling the CSF variations of p-tau and p-tau/Aβ_42_.

Decreased CSF Aβ_42_ levels are a well-established finding in AD and could reflect the aggregation of Aβ_42_ in brain tissue [27, 28], the formation of semisoluble Aβ_42_ oligomers [29], or even the binding of peptides in complexes that mask epitopes targeted by analytical assays [30]. Moreover, the hypothetical temporal changes of AD biomarkers presume that the CSF Aβ_42_ reduction begins in the pre-clinical stage [31]. However, repeated CSF measurements from symptomatic patients showed longitudinal stability of CSF Aβ_42_ levels, making this biomarker unsuitable for reflecting the underlying dynamics of amyloid metabolism over time [32]. On the other hand, amyR is a better predictor of abnormal cortical amyloid plaque burden in AD [11, 12], possibly because the addition of CSF Aβ_40_ in the ratio can correct for interindividual variability of amyloid production [33, 34]. However, another reasonable explanation may lie in a specific role of Aβ_40_ in AD. Biochemical studies have shown that Aβ_42_ has faster aggregation properties than other Aβ species and that Aβ_40_ monomers inhibit its aggregation [35–37], by preferentially binding to protofibrillar Aβ_42_ [38, 39]. Moreover, different ratios of Aβ_42_ to Aβ_40_ can result in different kinetics of amyloid fibrillization and aggregation. Chang and colleagues demonstrated that an equimolar Aβ_40_/Aβ_42_ sample generates oligomers with the highest neurotoxic effects on neuritic length, while an increase in the proportion of Aβ_40_ stabilizes the fibrillization pathway [40]. However, an Aβ_40_-dominant ratio causes changes in calcium dynamics, resulting in a higher elevation of intracellular calcium levels and neuronal apoptosis [41]. Thus, we may speculate that CSF levels of Aβ_40_ initially increase to quench the fibrillization pathway triggered by Aβ_42_ oligomerization; however, beyond a certain threshold, they may have toxic effects on neurons.

When we plotted the changes in CSF biomarkers as a function of amyR, we noticed different trends in CSF biomarkers variations. We observed that Aβ_42_ had the steepest decrease before the amyR-positivity cut-off and then encountered a plateau phase. In contrast, CSF p-tau and Aβ_40_ started increasing before the pathological threshold of amyR, but this increase was significantly more pronounced when amyR became pathological. This finding is consistent with the notion that cortical Aβ deposition precedes neocortical tau aggregation [34] and with previous studies reporting that the decrease in CSF Aβ_42_/Aβ_40_ ratio is followed by a large increase in CSF p-tau also in preclinical AD patients [42]. Indeed, we observed that a decrease of Aβ_42_ in the CSF is accompanied by an increase of tau phosphorylation but also that the increase of Aβ_40_ levels—which significantly affects amyR positivity—is accompanied by a steeper increase in CSF p-tau levels, which has recently been described to boost the spread of tau pathology in the brain [43].

In our cohort, we found that among A + T − patients, CSFAβ_42_ + /amyR + showed a higher probability of having pathological levels of p-tau/Aβ_42_, a well-known marker of AD-related burden, than CSFAβ_42_ + /amyR − . Considering the differences in CSF levels of Aβ_40,_ but not Aβ_42_, we might assume that the higher levels of Aβ_40_ in CSFAβ_42_ + /amyR + could concur with these results.

Unexpectedly, although the CSFAβ_42_ + /amyR − condition might be misinterpreted as an A − status if we relied on amyR more than CSF Aβ_42_, in our study, we found that pathological values of p-tau/Aβ_42_ can be present also in these patients. This highlights that amyR, commonly held as the best biomarker for detecting amyloid pathology [35], also shows suboptimal negative predictive power, suggesting that the added value of Aβ_40_ could lie beyond its Aβ_42_-corrective function, since correcting for inter-individual amyloid production variability should improve not only amyR specificity but also sensitivity.

The specific meaning of the altered CSF Aβ_40_ levels could be rooted in its significant correlation with the CSF levels of p-tau, which is also present under physiological conditions [44]. In our results, we observed a positive linear relationship between Aβ_40_ and p-tau in A − T − , CSFAβ_42_ + /amyR − , and CSFAβ_42_ + /amyR + , but not in A + T + . We cannot exclude that, alongside pathological Aβ_42_ levels, there may be a concomitant increase in CSF Aβ_40_ (as in CSFAβ_42_ + /amyR + patients) associated with a parallel increase in tau phosphorylation. Conversely, in our A + T + group tau pathology seems to proceed independently from amyloid pathology as no association was found between amyloid and tau CSF biomarkers. In line with our results, it has been recently demonstrated that the Aβ-induced increase in CSF p-tau levels might play a key role in initiating tau aggregation and spreading in early AD, while local tau seeding and auto-replication predominate once soluble p-tau concentrations reach a plateau in AD dementia [43].

### Main findings in FDG-PET metabolic pattern

To support our hypothesis that CSFAβ_42_ + and amyR + hold different meanings in AD pathophysiology, we considered patterns of brain glucose metabolism. Indeed, specific FDG-PET hypometabolic patterns have been demonstrated to predict AD dementia [15–17] and may detect additional differences between CSFAβ_42_ + /amyR − and CSFAβ_42_ + /amyR + .

First, when comparing FDG-PET scans from CSFAβ_42_ + /amyR − patients and controls, we found a pattern of cortical hypometabolism encompassing the parietal and frontotemporal regions. Thus, the decrease in CSF Aβ_42_ alone may be associated with synaptic dysfunction and reduced brain glucose uptake in areas typically involved in AD [45, 46]. However, other crucial factors (i.e., such as endothelial dysfunction, neuroinflammation, and astrocytic impairment) could also have influenced synaptic functioning, especially in the very early stages of the disease, before overt amyloid pathology occurs [47–49].

In contrast, patients with CSFAβ_42_ + /amyR + showed further involvement of the frontal regions, configuring an FDG-PET pattern indistinguishable from A + T + . Previous studies have shown that soluble Aβ_40_, but not Aβ_42_, extracted from the frontal cortex of patients with AD gradually increases along with the progression of Braak scores, suggesting a specific link between Aβ_40_ levels and the progression of tau pathology [50]. Moreover, a positive correlation between soluble tau levels and hypometabolism in the frontal regions has also been reported [51]. Our findings suggest that amyR positivity, which in our cohort is determined by the co-presence of decreased Aβ_42_ and increased Aβ_40_ levels, is associated with widespread brain hypometabolism, and probably with the spread of tau pathology, even in the absence of elevated tau proteins in the CSF [52].

The lack of detection of elevated levels of CSF p-tau_181_ in our cohort does not exclude a possible increase of other p-tau isoforms (e.g., p-tau_217_ or p-tau_231_). Moreover, a threshold-based evaluation of CSF p-tau, as for other fluid biomarkers, can have limitations tied to the risk of false negative results, whose occurrence also encompasses pre-analytical (e.g., collection techniques, handling of samples, storage conditions) and analytical variables (e.g., accuracy in sample processing, assay sensitivity) [53]. On the other hand, there may be other yet-to-be elucidated pathophysiological processes underlying a possible mismatch between parenchymal and CSF evidence of tau pathology. Considering all these limitations, a multimodal approach combining imaging and CSF biomarkers could be advisable.

Our results show that among patients classified as A + T − , there is a group with reduced Aβ_42_ and low Aβ_40_ (CSFAβ_42_ + /amyR −) that differ from healthy controls for showing typical AD patterns of cerebral hypometabolism, and a second group with reduced Aβ_42_ and high Aβ_40_ (CSFAβ_42_ + /amyR +) showing widespread reduction of brain glucose uptake, undistinguishable from biologically defined AD patients (A + T +).

## Limitations

To our knowledge, this study is the first to directly compare CSFAβ_42_ + /amyR − and CSFAβ_42_ + /amyR + patients, as well as the first to examine differences and overlap between them, healthy controls, and A + T + in terms of CSF p-tau, AD-related burden, and brain FDG-PET metabolic patterns. However, because of its cross-sectional design, we could not investigate temporal connection between different biomarkers alteration or evaluate whether higher CSF Aβ_40_ levels, along with pathological CSF Aβ_42_, may affect the rapidity of cognitive decline.

Furthermore, fluid biomarkers, represented on a continuous scale, are commonly used in a dichotomous way, applying cut-offs whose thresholds become crucial in clinical practice. Such thresholds are not always available for every biomarker measure, and a global effort of standardization is needed to increase accuracy and reproducibility of these findings. Further investigations using amyloid-PET or tau-PET would be useful to verify our results on the prevalence of pathological AD-related burden across patient groups—which we evaluated indirectly via the CSF p-tau/Aβ_42_ ratio—and to assess any causal connections between the alteration of Aβ_42_, Aβ_40_, and p-tau in the CSF and parenchymal amyloid and tau deposition. Finally, extending the analysis by comparing blood-based and CSF biomarkers could be useful to confirm these relationships and to assess their extensibility for diagnostic and prognostic purposes.

## Conclusions

Fluid biomarkers are nowadays being introduced into clinical routine practice, positing challenges on their correct use for either supporting or excluding AD diagnosis.

Patients with cognitive symptoms and decreased CSF Aβ_42_ without pathological amyR nor increased p-tau levels could, under certain circumstances, be essentially considered non-AD, but this conclusion can be hard to reach when clinical presentation and FDG-PET pattern are highly suggestive of AD. Our results suggest that decreased CSF Aβ_42_ alone could detect Alzheimer’s pathology, since these patients are distinguished from controls by reduced FDG uptake in brain regions typical of AD, and some of them also show pathological measures of AD-related burden.

On the other hand, the increase in CSF Aβ_40_ levels, traced by pathological amyR, associates with higher levels of soluble hyperphosphorylated tau, higher AD-related burden, and a widespread pattern of FDG hypometabolism.

Considering the many experimental novel drugs that see Aβ and tau proteins as therapeutic targets, the specific meaning of different amyloid CSF biomarkers should be considered to support a more accurate selection of patients and search for the best time window for treatment.

### Supplementary Information

**Additional file 1.** Flowcharts summarizing patients’ enrolment (A) and control group selection (B) procedures for the CSF study.

**Additional file 2.** Flowcharts summarizing patients’ enrolment (A) and control group selection (B) procedures for the FDG-PET substudy.

**Additional file 3.** Scatter Plots showing correlations (Spearman’s rho) between CSF p-tau and different amyloid biomarkers (Aβ_42_, Aβ_40_, amyR) in A-T-, CSFAβ_42_+/amyR-, CSFAβ_42_+/amyR+ and A+T+.

**Additional file 4.** Demographical, clinical and biomarkers data from FDG-PET substudy.

**Additional file 5.** Numerical results of SPM comparisons of FDG uptake in CG vs. CSFAβ42+/amyR-, CG vs. CSFAβ42+/amyR+ and CG vs. A+T+.

## Data Availability

The dataset analyzed in this study is available from the corresponding author upon reasonable request.

## Abbreviations

AD: Alzheimer’s disease
Aβ: Amyloid-β
amyR: Amyloid ratio
BA: Brodmann area
CSF: Cerebrospinal fluid
CT: Computed tomography
FDG: Fludeoxyglucose
MRI: Magnetic resonance imaging
MMSE: Mini-Mental State Examination
PET: Positron emission tomography
PCR: Polymerase chain reaction
SPM: Statistical parametric mapping
