# Supplementary material for: Different associations between amyloid-βeta 42, amyloid-βeta 40, and amyloid-βeta 42/40 with soluble phosphorylated-tau and disease burden in Alzheimer’s disease: a cerebrospinal fluid and fluorodeoxyglucose-positron emission tomography study
Source: Alzheimers Res Ther. 2023 Aug 30;15:144. doi: 10.1186/s13195-023-01291-w (PMC10466826; doi:10.1186/s13195-023-01291-w)

**Additional File 1: Flowcharts summarizing patients’ enrolment (A) and control group selection (B) procedures for the CSF study.**


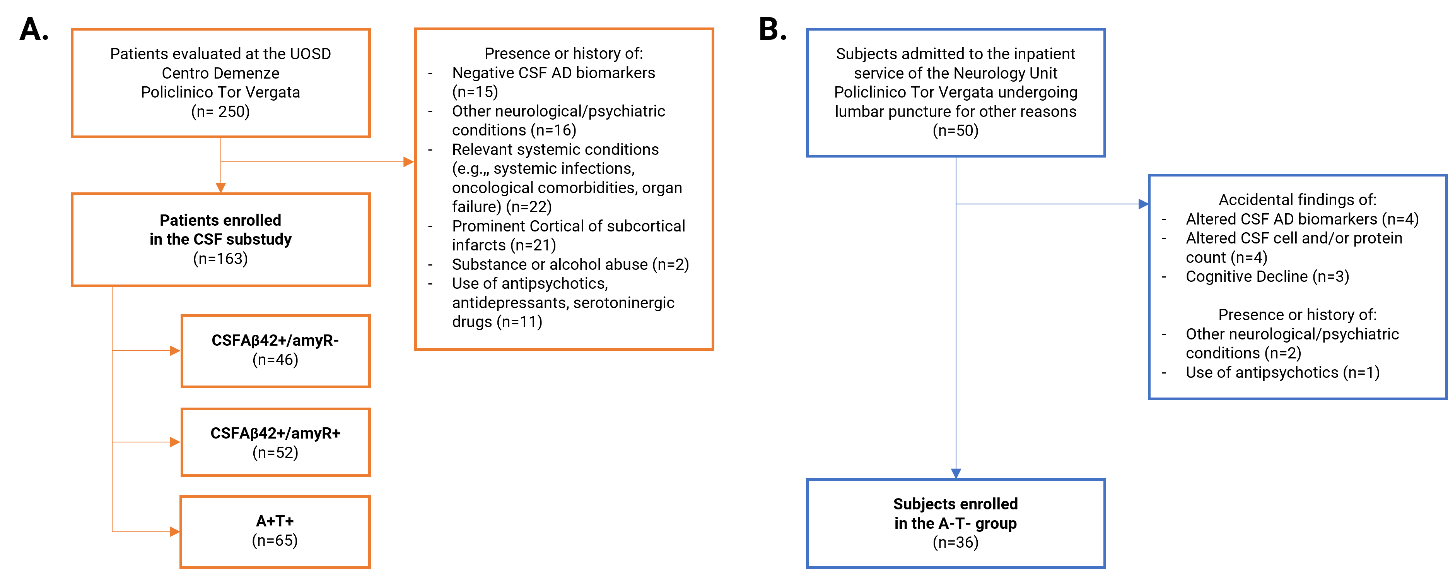

Supplement: Supplementary file 1 — Additional file 1. Flowcharts summarizing patients’ enrolment (A) and control group selection (B) procedures for the CSF study. [file 13195_2023_1291_MOESM1_ESM.docx]
