# Supplementary material for: Different associations between amyloid-βeta 42, amyloid-βeta 40, and amyloid-βeta 42/40 with soluble phosphorylated-tau and disease burden in Alzheimer’s disease: a cerebrospinal fluid and fluorodeoxyglucose-positron emission tomography study
Source: Alzheimers Res Ther. 2023 Aug 30;15:144. doi: 10.1186/s13195-023-01291-w (PMC10466826; doi:10.1186/s13195-023-01291-w)
Supplement: Supplementary file 3 — Additional file 3. Scatter Plots showing correlations (Spearman’s rho) between CSF p-tau and different amyloid biomarkers (Aβ42, Aβ40, amyR) in A-T-, CSFAβ42+/amyR-, CSFAβ42+/amyR+ and A+T+. [file 13195_2023_1291_MOESM3_ESM.docx]

**Additional File 3:**


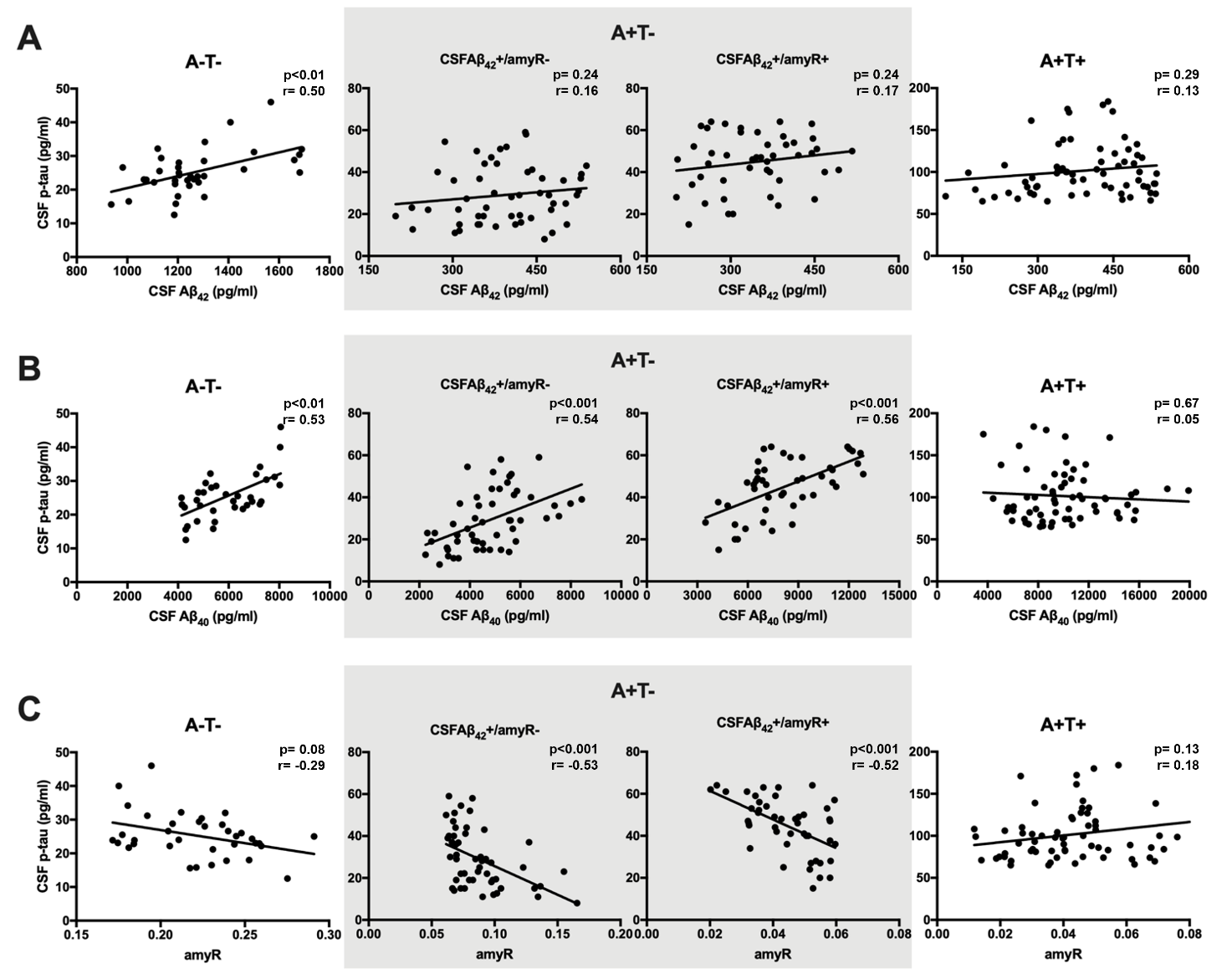


Scatter Plots showing correlations (Spearman’s rho) between CSF p-tau and different amyloid biomarkers (Aβ_42_, Aβ_40_, amyR) in A-T-, CSFAβ_42_+/amyR-, CSFAβ_42_+/amyR+ and A+T+.
