# Supplementary material for: Different associations between amyloid-βeta 42, amyloid-βeta 40, and amyloid-βeta 42/40 with soluble phosphorylated-tau and disease burden in Alzheimer’s disease: a cerebrospinal fluid and fluorodeoxyglucose-positron emission tomography study
Source: Alzheimers Res Ther. 2023 Aug 30;15:144. doi: 10.1186/s13195-023-01291-w (PMC10466826; doi:10.1186/s13195-023-01291-w)
Supplement: Supplementary file 4 — Additional file 4. Demographical, clinical and biomarkers data from FDG-PET substudy. [file 13195_2023_1291_MOESM4_ESM.docx]

**Additional File 4: Demographical, clinical and biomarkers data from FDG-PET substudy**

|  | **CSFAβ42+/amyR-**  **(n=9)** | **CSFAβ42+/amyR+**  **(n=17)** | **A+T+**  **(n=20)** | **CG**  **(n=24)** |
| --- | --- | --- | --- | --- |
| **Age (years)** | 68.01±6.98 | 71.34±8.91 | 68.91±9.56 | 66.56±10.44 |
| **Male (%)** | 44.4% | 58.8% | 45% | 41.7% |
| **MMSE** | 19.13±6.14 | 20.66±5.90 | 20.37 ± 4.92 | n.a. |
| **APOE4 (%)** | 55.5% | 23.5% | 50% | n.a. |
| **CSF Aβ_42_ (pg/ml)** | 401.39±83.56 | 388.94±83.68 | 392.73±105.62 | n.a. |
| **CSF Aβ_40_ (pg/ml)** | 5617.39±1340.56 | 8331.59±2739.53 | 10119.85±3397.07 | n.a. |
| **CSF p-tau (pg/ml)** | 40.18±12.82 | 43.47±14.06 | 103.77±30.93 | n.a. |
| **CSF t-tau (pg/ml)** | 174.73±72.21 | 227.76±67.86 | 663.42±263.47 | n.a. |
| **CSF p-tau/Aβ_42_** | 0.10±0.04 | 0.13±0.05 | 0.28±0.11 | n.a. |
| **CSF p-tau/Aβ_42_ > 0.086 (n)** | 5 | 14 | 20 | n.a. |
| **CSF p-tau/Aβ_42_ > 0.122 (n)** | 4 | 9 | 20 | n.a. |
| **Pattern of FDG-PET** | | | | |
| **Typical of AD (n)** | 7 | 14 | 16 | 0 |
| **Possible AD (n)** | 0 | 1 | 3 | 0 |
| **Normal (n)** | 2 | 2 | 1 | 24 |

Additional File 4 legend: Data are presented as mean ± standard deviation, percentages or counts, as applicable. CSF, cerebrospinal fluid; MMSE, Mini-Mental State Examination; FDG, Fluorodeoxyglucose; n.a., not available.
