# Supplementary material for: Different associations between amyloid-βeta 42, amyloid-βeta 40, and amyloid-βeta 42/40 with soluble phosphorylated-tau and disease burden in Alzheimer’s disease: a cerebrospinal fluid and fluorodeoxyglucose-positron emission tomography study
Source: Alzheimers Res Ther. 2023 Aug 30;15:144. doi: 10.1186/s13195-023-01291-w (PMC10466826; doi:10.1186/s13195-023-01291-w)
Supplement: Supplementary file 5 — Additional file 5. Numerical results of SPM comparisons of FDG uptake in CG vs. CSFAβ42+/amyR-, CG vs. CSFAβ42+/amyR+ and CG vs. A+T+. [file 13195_2023_1291_MOESM5_ESM.docx]

**Additional File 5. Numerical results of SPM comparisons of FDG uptake in CG vs. CSFAβ42+/amyR-, CG vs. CSFAβ42+/amyR+ and CG vs. A+T+.**

| **Analysis** | **Cluster level** | | | | | **Voxel level** | | |  |  |
| --- | --- | --- | --- | --- | --- | --- | --- | --- | --- | --- |
|  | **cluster p (FWE-corr)** | **cluster p (FDR-corr)** | **Cluster extent** | **Cortical Region** | **Z score of maximum** | **Talairach coordinates** | **Cortical region** | **BA** |  |  |
| **CG**  **vs**  **CSFAβ42+/amyR-** | 0.000 | 0.000 | 31175 | L Parietal Lobe | Inf. | -36, -74, 38 | Precuneus | 19 |  |  |
|  |  | | | L Temporal Lobe | Inf. | -52, -56, -10 | Middle Temporal Gyrus | 37 |  |  |
|  |  |  |  | L Parietal Lobe | 7.63 | -48, -48, 44 | Inferior Parietal Lobule | 40 |  |  |
|  | 0.001 | 0.000 | 1795 | L Frontal Lobe | 3.81 | -26, 10, 58 | Middle Frontal Gyrus | 6 |  |  |
|  |  | | | L Frontal Lobe | 3.61 | -30, 26, 46 | Middle Frontal Gyrus | 8 |  |  |
|  |  |  |  | L Frontal Lobe | 3.43 | -48, 30, 16 | Inferior Frontal Gyrus | 46 |  |  |
| **CG**  **vs**  **CSFAβ42+/amyR+** | 0.000 | 0.000 | 17523 | L Occipital Lobe | 5.77 | -8, -66, 24 | Precuneus | 31 |  |  |
|  |  | | | L Parietal Lobe | 4.96 | -8, -48, 34 | Precuneus | 31 |  |  |
|  |  |  |  | L Parietal Lobe | 4.93 | -46, -52, 46 | Inferior Parietal Lobule | 40 |  |  |
|  |  |  |  | L Temporal Lobe | 4.75 | -54, -24, -4 | Middle Temporal Gyrus | 21 |  |  |
|  | 0.045 | 0.022 | 1527 | L Frontal Lobe | 4.27 | 18, 60, 12 | Superior Frontal Gyrus | 10 |  |  |
|  |  | | | L Frontal Lobe | 3.96 | -18, 56, 22 | Superior Frontal Gyrus | 10 |  |  |
|  |  |  |  | L Frontal Lobe | 3.4 | -26, 40, 36 | Middle Frontal Gyrus | 9 |  |  |
| **CG**  **vs**  **A+T+** | 0.000 | 0.000 | 22058 | R Parietal Lobe | 6.03 | 42, -60, 34 | Angular gyrus | 39 |  |  |
|  |  | | | L Temporal Lobe | 5.86 | -50, -20, -28 | Inferior Temporal Gyrus | 20 |  |  |
|  |  |  |  | L Temporal Lobe | 6.31 | -52, -42, -18 | Inferior Temporal Gyrus | 37 |  |  |
|  | 0.002 | 0.001 | 3139 | L Frontal Lobe | 4.97 | -26, 12, 58 | Middle Frontal Gyrus | 6 |  |  |
|  |  | | | R Frontal Lobe | 4.55 | 26, 40, 42 | Superior Frontal Gyrus | 8 |  |  |
|  |  |  |  | L Frontal Lobe | 4.40 | -28, 28, 46 | Middle Frontal Gyrus | 8 |  |  |

Additional File 5 legend: In the ‘cluster level’ section (left), the number of voxels, corrected p-value of significance, and cortical region where the voxel is found are all reported for each significant cluster. In the ‘voxel level’ section, all the coordinates of the correlation sites (with the Z-score of the maximum correlation point), the corresponding cortical region and BA are reported for each significant cluster. (CG, control group; L, left; R, right; BA, Brodmann area). When the maximum correlation is achieved outside the gray matter, the nearest gray matter (within a range of 5 mm) is indicated by the corresponding BA.
